# Plasma p-Tau217 and amyloid-β oligomers as complementary biomarkers for differential diagnosis, comorbidity detection and disease monitoring in idiopathic normal pressure hydrocephalus

**DOI:** 10.1186/s12987-026-00784-8

**Published:** 2026-03-05

**Authors:** Ko Horikoshi, Madoka Nakajima, Masakazu Miyajima, Ryo Miyahara, Koichiro Sakamoto, Kaito Kawamura, Chihiro Akiba, Chihiro Kamohara, Ikuko Ogino, Taiji Tsunemi, Kostadin Karagiozov, Akihide Kondo

**Affiliations:** 1 Department of Neurosurgery, Juntendo University School of Medicine, 2-1-1, Hongo, Bunkyo-ku, Tokyo, 113-8421 Japan; 2 Department of Neurosurgery, Juntendo Tokyo Koto Geriatric Medical Center, Tokyo, Japan; 3 Research Institute for Diseases of Old Age, Juntendo University School of Medicine, Tokyo, Japan; 4 Department of Neurology, Juntendo University School of Medicine, Tokyo, Japan

**Keywords:** Idiopathic normal pressure hydrocephalus, Alzheimer’s disease, CSF, Plasma, Biomarker, p-Tau, AβO

## Abstract

**Background:**

Idiopathic normal pressure hydrocephalus (iNPH) overlaps clinically and pathologically with Alzheimer’s disease (AD), making differential diagnosis and prognostication challenging. The identification of reliable and minimally invasive plasma biomarkers would have major clinical implications for distinguishing iNPH from AD and for monitoring disease course and surgical outcomes.

**Methods:**

We conducted three studies: (Study 1) cross‑sectional comparisons of plasma phosphorylated tau 217 (p‑Tau217) and amyloid-β oligomers (AβO) among healthy controls (HCs; *n* = 22), iNPH (*n* = 76), and AD (*n* = 23) groups, accompanied by receiver operating characteristic (ROC) analysis; (Study 2) correlation analyses within preoperative iNPH group to assess associations between biomarkers and clinical indices, as well as two‑group comparisons between iNPH group with and without AD pathology stratified by CSF p‑Tau181 > 30 pg/mL; and (Study 3) longitudinal pre- and postoperative assessments following shunt surgery.

**Results:**

In the three‑group analyses, plasma p‑Tau217 effectively discriminated iNPH from AD (AUC = 0.79, 95% = CI 0.700–0.882), whereas discrimination between HCs and iNPH was poor (AUC = 0.48). Plasma AβO moderately discriminated HCs from iNPH (AUC = 0.71, 95% CI = 0.567–0.848). In the preoperative iNPH group, CSF and plasma p‑Tau217 showed a strong correlation (ρ = 0.736, *p* < 0.001), whereas CSF and plasma AβO showed no significant correlation (ρ = −0.047, *p* = 0.689). Among iNPH group with AD pathology (CSF p-Tau181 > 30 pg/mL), plasma p-Tau217 was higher, plasma AβO was lower, and Frontal Assessment Battery (FAB) scores were reduced compared with those without AD pathology. For detecting coexistent AD pathology in iNPH group, AUCs were modest for plasma p‑Tau217 (0.64), AβO (0.63), and p‑Tau217/AβO ratio (0.66). Postoperatively, iNPH grading scale (GS), Mini-Mental State Examination (MMSE), and FAB scores improved significantly; CSF and plasma AβO levels decreased, CSF p‑Tau217 increased, whereas plasma p‑Tau217 levels remained stable.

**Conclusions:**

Plasma p-Tau217, reflecting AD-related pathology, and plasm AβO, reflecting disease stage, provide complementary information for distinguishing iNPH from AD, assessing comorbidity of AD pathology in iNPH and evaluating postoperative changes. Their combined measurement may support differential diagnosis, aid the preoperative identification of concomitant AD pathology in iNPH, and serve as a minimally invasive screening and triage tool as well as a means of postoperative monitoring, while acknowledging that discrimination between HCs and iNPH at individual level remains limited.

**Trial registration:**

Not applicable.

**Supplementary Information:**

The online version contains supplementary material available at 10.1186/s12987-026-00784-8.

## Background

Idiopathic normal pressure hydrocephalus (iNPH) is characterized by the clinical triad of gait disturbance, cognitive impairment, and urinary incontinence with ventriculomegaly, most often affecting older adults. It reflects disturbed cerebrospinal fluid (CSF) absorption, and symptoms can improve after shunt surgery, making iNPH a treatable cause of dementia [1, 2].

Clinical overlap between iNPH and Alzheimer’s disease (AD) is substantial in older adults, making differential diagnosis challenging. Autopsy studies have reported that more than half of clinically diagnosed iNPH cases harbor concomitant AD pathology, indicating that the two conditions substantially overlap [3]. Moreover, several reports have proposed that some iNPH cases may lie along the early stages of AD-related pathology [4].

CSF biomarkers in iNPH have attracted growing attention. While decreased CSF amyloid-β (Aβ) 42 and increased tau species are typical for AD, findings in iNPH have been inconsistent, possibly due to altered CSF dynamics and heterogeneous pathophysiology. Some studies have shown that CSF Aβ42 toxic conformer ratios may reflect prognosis after shunting [5–8]. Tau is a microtubule-associated protein; its pathological phosphorylation contributes to tau aggregation and neurodegeneration. Among phosphorylated tau, the 217 species (p-Tau217) has emerged as especially informative for AD, in some studies outperforming p-Tau181 and rising early along the amyloid-first cascade [9–11]. Amyloid-β oligomers (AβO) are highly neurotoxic and distinct from monomers and plaques. Notably, AβO is elevated in CSF in AD and correlates with cognitive function; in iNPH, CSF AβO levels are higher than in controls and decline after shunting, with baseline levels tentatively linked to postoperative cognitive outcomes [12, 13].

Because blood sampling is minimally invasive, establishing plasma biomarkers for iNPH could potentially aid diagnosis and prognosis. Research on plasma p-Tau217 and AβO in iNPH remains limited. We therefore compared these markers across healthy controls (HCs), iNPH, and AD, and evaluated preoperative and postoperative changes as well as their associations with clinical features and CSF biomarkers.

## Methods

### Study population

We enrolled probable iNPH patients (*n* = 76), with diagnosis based on clinical features, imaging findings and the tap test in accordance with the Japanese iNPH clinical guidelines [1, 14]. Responses to tap test were evaluated using standardized assessments of gait, cognition, and urinary symptoms before and after CSF removal, including the iNPH grading scale (GS) as appropriate. Major exclusion criteria included secondary NPH and other major neurodegenerative diseases. AD patients (*n* = 23) were diagnosed according to the 2011 National Institute on Aging and Alzheimer’s Association (NIA–AA) criteria [15], supported by imaging and/or CSF biomarkers (Aβ42/40, p‑Tau181), and did not exhibit imaging features characteristic of iNPH (e.g., ventriculomegaly with disproportionately enlarged subarachnoid-space hydrocephalus). Cognitive function was routinely assessed using the Mini Mental State Examination (MMSE). HCs (*n* = 22) had normal brain structure on MRI, no evidence of cognitive decline, and no CSF-related disorders.

### Study design

#### Study 1

Plasma p‑Tau217 and AβO were measured and compared across the HCs, iNPH, and AD groups. Pairwise comparisons and receiver operating characteristic (ROC) analyses assessed group discrimination.

#### Study 2

In the preoperative iNPH group, clinical scores (Evans’ index, iNPH grading scale [16], MMSE, and Frontal Assessment Battery (FAB) were evaluated. The iNPH GS rates gait disturbance (G), cognitive impairment (D), and urinary dysfunction (U) using ordinal scores, whereas MMSE provides a global cognitive screening score and FAB assesses frontal executive function. CSF biomarkers (p‑Tau181, Aβ42, Aβ40, Aβ42/40, p‑Tau217, and AβO) and plasma biomarkers (p‑Tau217, AβO) were analyzed for correlations. The iNPH group was stratified by CSF p‑Tau181 > 30 pg/mL to define iNPH with AD pathology and without [17], and group comparisons and ROC analyses were performed.

Concomitant AD pathology in iNPH was operationally defined as preoperative CSF p‑Tau181 > 30 pg/mL. This cutoff was selected based on prior outcome-based evidence in iNPH showing that p‑Tau181 > 30 pg/mL is associated with a shorter durability of postoperative cognitive improvement after shunt surgery, consistent with underlying AD pathology [17]. Although the CSF Aβ42/40 ratio is widely accepted as a core AD biomarker, it was not used for primary stratification because CSF Aβ measures can be reduced in iNPH due to CSF flow disturbances [18], and CSF Aβ42/40 was not routinely available in our clinical practice during the study period. In addition, to examine whether the biomarkers’ alterations observed in the iNPH with AD pathology group merely reflected Alzheimer’s disease–related changes, an additional comparison was performed between the iNPH with AD pathology group and the AD group using the same plasma biomarkers.

#### Study 3

Longitudinal preoperative and postoperative analyses after shunt surgery included 75 patients from the iNPH group treated by lumbo‑peritoneal shunt (LPS) surgery with both CSF and plasma sampling. The postoperative evaluation period ranged from 1 to 42 months, with a mean of 10.0 ± 8.4 months. Clinical scores and biomarkers (p-Tau217 and AβO) changes pre- and postoperatively were evaluated and compared between the iNPH subgroups with and without AD pathology groups.

### CSF and plasma collection

In the preoperative iNPH group, CSF was collected at the tap test (L3/4 or L4/5). Postoperative CSF was obtained from the LP shunt-valve reservoir. To avoid contamination from residual fluid within the reservoir, the initial 2 mL of CSF was discarded, and the subsequent sample was collected for measurement. CSF and plasma were centrifuged at 4 °C, at 1,690 g for 15 min, aliquoted into polypropylene tubes, and stored at − 80 °C. Plasma was processed according to standard procedures, with a single freeze–thaw cycle.

### Biomarker assays

The biomarkers were measured using the following immunoassays: p-Tau181 (T1008, Nipro); Aβ40 and Aβ42 (INNOTEST, Fujirebio); AβO (Fujifilm Wako). BAN50 antibody was used for both capture and detection; this assay specifically detects AβO containing 10–20 monomers (corresponding to 40–200 kDa) and does not detect monomers, dimers, trimers, tetramers, or hexamers [13, 19]. Luminescence-based measurements were used to detect the target AβO^10–20^ given that these represent < 1% of total AβO^10–20^. Therefore, we used a 96-well microplate reader (SpectraMax L; Molecular Devices Japan, Tokyo, Japan) and its accompanying software (SoftMax Pro 5.4.8). P-Tau217 (S-PLEX Human Tau [pT217], Meso Scale Discovery). S-PLEX assays were performed according to the manufacturer’s instructions, including blocking, capture, TURBOBOOST detection, Enhance E1–E3, TURBOTAG labeling, and readout on the QuickPlex SQ 120 platform. Calibration curves were fitted with a 4-parameter logistic (1/Y²) model. All samples were measured in duplicate, and results were accepted only when duplicate coefficients of variation met prespecified quality criteria [20].

### Statistics

All statistical analyses were performed using IBM SPSS Statistics version 29.0.2.0 (IBM Corp., Armonk, NY, USA). Descriptive statistics are presented as mean ± SD or median [IQR], as appropriate. Group comparisons were conducted using Welch’s t test, chi-square test, Kruskal–Wallis test, or Mann–Whitney U test. Correlations were assessed with Spearman’s correlation coefficient. ROC analyses were performed to calculate the area under the curve (AUC, 95% CI); optimal cut-off values were determined by maximizing Youden’s index, and sensitivity and specificity were reported. Paired data were analyzed with the Wilcoxon signed-rank test (two-sided, Pratt method). A two-tailed p value < 0.05 was considered statistically significant (*p < 0.05; **p < 0.01; ***p < 0.001).

To evaluate whether postoperative changes differed between iNPH patients with and without concomitant AD pathology (Study 3), we examined the interaction between time (preoperative vs. postoperative) and group (with vs. without AD pathology) using a two-way mixed repeated-measures analysis of covariance (ANCOVA). Because the two subgroups were not fully age-matched, age was included as a covariate in the repeated-measures ANCOVA model.

## Results

### Study 1: Comparisons between the iNPH group and the HCs and AD groups

**Fig. 1:**
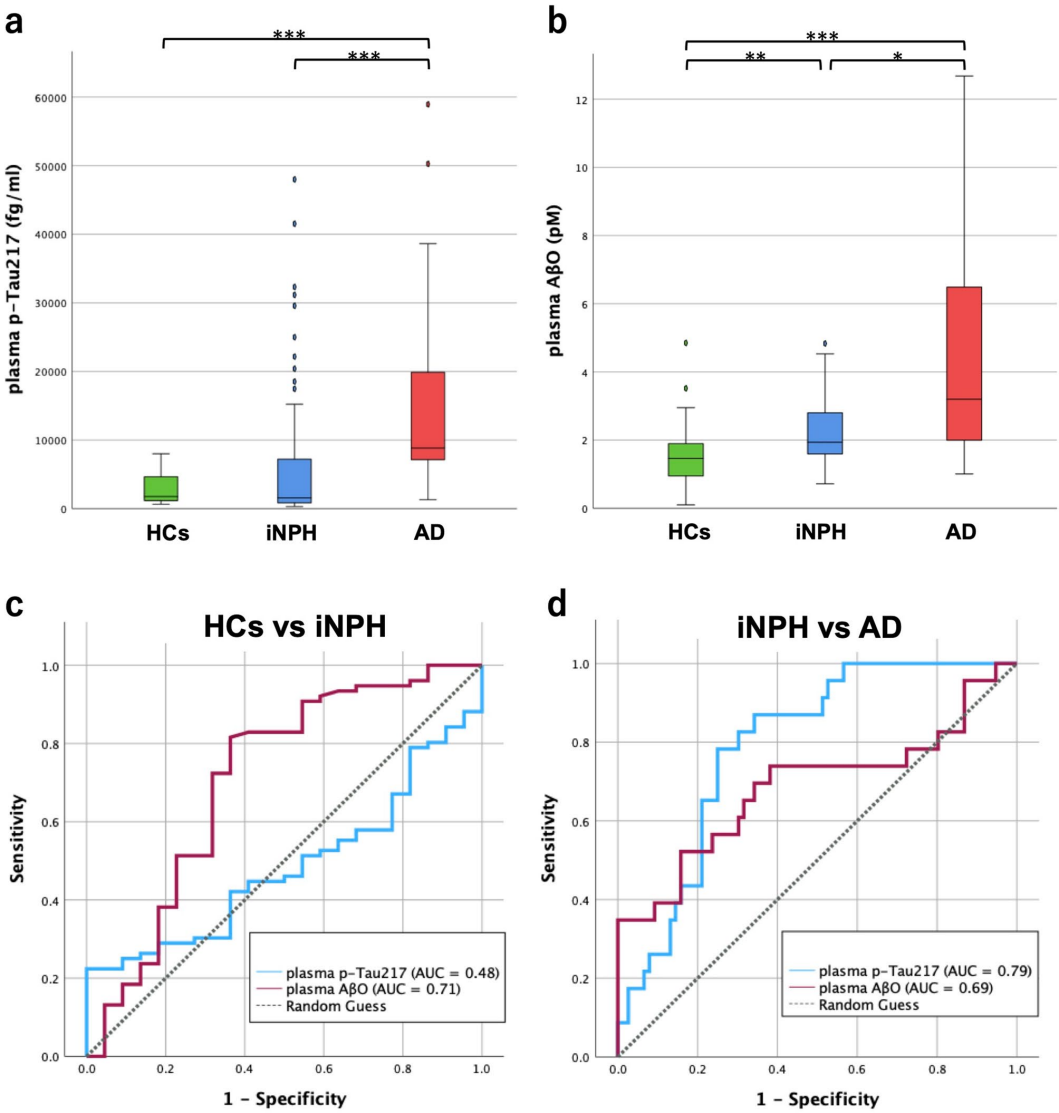
Box plots and ROC analyses for discrimination in each group by plasma biomarkers. Box-and-whisker plots show plasma p-Tau217 (**a**) and AβO (**b**) concentrations in the HCs (green), iNPH (blue) and AD (red) group. Median, IQR, and whiskers (1.5 × IQR) are displayed. Plasma p-Tau217 levels were significantly higher in the AD group than both the HCs and iNPH groups (***p < 0.001, Kruskal–Wallis test followed by pairwise Mann–Whitney U tests). Plasma AβO levels were significantly higher in the AD group than the iNPH groups (*p = 0.013) and higher in the iNPH group than the HCs groups (**p = 0.006). ROC curves show each pairwise (**c**: HCs vs. iNPH, **d**: iNPH vs. AD) discrimination using p‑Tau217 (blue) and AβO (purple). Curves are plotted as sensitivity versus 1–specificity; the diagonal grey dashed line indicates random guess. The AUC values are shown in the in‑panel keys: HCs vs. iNPH—p‑Tau217 (0.48), AβO (0.71); iNPH vs. AD—p‑Tau217 (0.79), AβO (0.69). Abbreviations: p-Tau217, phosphorylated tau 217; AβO, amyloid-β oligomers; HCs, healthy controls; iNPH, idiopathic normal pressure hydrocephalus; AD, Alzheimer’s disease; IQR, interquartile range; ROC, receiver operating characteristic; AUC, area under the curve

In 3-group comparisons (Tables 1 and 2; Fig. 1), there were no significant differences in age or sex distribution among the three groups, and MMSE was significantly lower in AD than in iNPH group (median [IQR]: 16.0 [12.0–23.5] vs. 25.0 [22.0–28.0], ***p < 0.001) (Table 1). Plasma p-Tau217 levels were 2,970 ± 2,515 (mean ± SD) fg/mL in HCs, 6,188 ± 9,970 fg/mL in iNPH, and 15,963 ± 15,694 fg/mL in AD. Although the overall difference among groups was significant (Kruskal–Wallis test, ***p < 0.001), post hoc pairwise comparisons showed no significant difference between HCs and iNPH (*p* = 0.951) but showed significant differences between iNPH vs. AD and AD vs. HCs (***p < 0.001). By contrast, plasma AβO differed across HCs (1.61 ± 1.13 pM), iNPH (2.27 ± 1.00 pM), and AD (4.42 ± 3.38 pM) (Kruskal–Wallis test, ***p < 0.001) and showed significant differences in all pairwise comparisons. ROC analyses (Table 2; Fig. 1) demonstrated that p-Tau217 discriminated iNPH from AD with an AUC of 0.79 (95% CI, 0.700–0.882) at a cutoff of 6,821 fg/mL (sensitivity 78.3%, specificity 75.0%), whereas HCs vs. iNPH discrimination was poor (AUC = 0.48). For AβO, HCs vs. iNPH yielded an AUC of 0.71 (95% CI, 0.567–0.848) at a cutoff of 1.53 pM (sensitivity 81.8%, specificity 63.6%). Pairwise comparison and ROC analysis between AD vs. HCs showed in supplementary Fig. 1.

**Table 1:** Baseline across 3-group

	HCs (*n* = 22)	iNPH (*n* = 76)	AD (*n* = 23)	*p*-value
Age (mean ± SD)	75.7 ± 8.7	78.0 ± 6.4	78.5 ± 8.4	0.788
Male (n, %)	10 (45.5%)	42 (55.3%)	11 (47.8%)	0.862
MMSE (median [IQR])		25 [22–28]	16 [12–23.5]	< 0.001***
Plasma biomarker (mean ± SD)				
p-Tau217 (fg/mL)	2970 ± 2515	6188 ± 9970	15,963 ± 15,694	< 0.001***
AβO (pM)	1.61 ± 1.13	2.27 ± 1.00	4.42 ± 3.38	< 0.001***

P-values across 3-group (the HCs, iNPH and AD group) of Welch’s t test (age), chi-square test (male), Mann–Whitney U test (MMSE), Kruskal–Wallis test (biomarkers) are shown in table. Significance in multiple comparison is shown as *p < 0.05; **p < 0.01; ***p < 0.001

**Table 2:** Pairwise comparisons and ROC analyses by plasma biomarkers

	HCs vs. iNPH	iNPH vs. AD	AD vs. HCs
plasma *p*-Tau217			
p-value	0.951	< 0.001***	< 0.001***
AUC	0.48	0.79	0.88
95% CI	0.365–0.604	0.700–0.882	0.780–0.979
Cutoff (fg/mL)	8056	6821	8074
Sensitivity	0.224	0.783	0.652
Specificity	1.000	0.750	1.000
plasma AβO			
p-value	0.006**	0.013 *	< 0.001***
AUC	0.71	0.69	0.82
95% CI	0.567–0.848	0.541–0.831	0.694–0.938
Cutoff (pM)	1.53	3.15	2.29
Sensitivity	0.818	0.522	0.739
Specificity	0.636	0.842	0.818

Pairwise comparisons (HCs vs. iNPH, iNPH vs. AD, AD vs. HCs) and ROC analyses for plasma p‑Tau217 and AβO are shown in table. Between‑group p‑values were calculated with Dunn–Bonferroni post-hoc analysis. ROC output includes AUC (95% CI), optimal cut‑off (Youden index), sensitivity, and specificity. Significance in multiple comparison is shown as *p < 0.05; **p < 0.01; ***p < 0.001

### Study 2: Comparisons between the iNPH with and without AD pathology groups

**Fig. 2:**
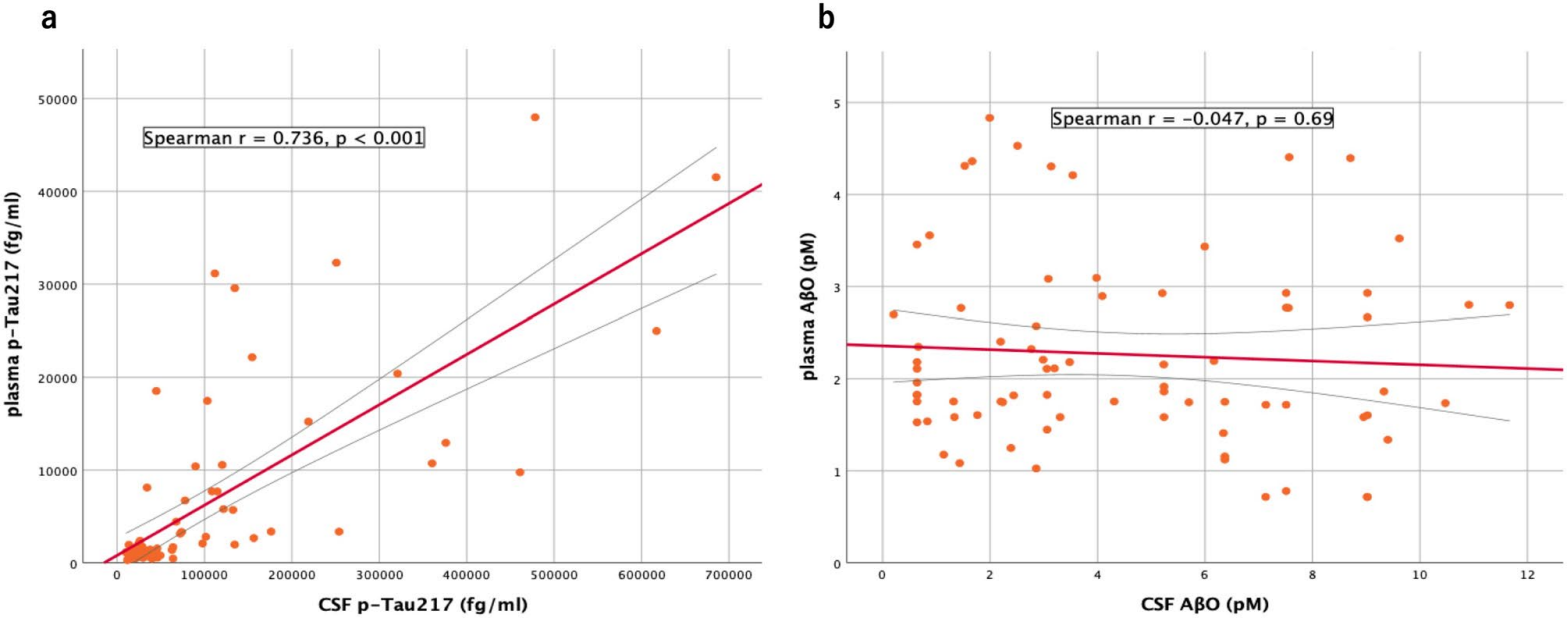
CSF–plasma correlations in preoperative iNPH. Scatter plots show the correlations between CSF and plasma concentrations in the preoperative iNPH group. Red solid lines show least‑squares fits with grey 95% confidence bands; statistics were evaluated with Spearman’s rank correlation (two‑sided). A strong positive correlation was observed for p‑Tau217 (**a**) (*r* = 0.736, *p* < 0.001), whereas AβO (**b**) showed no significant CSF–plasma correlation (*r* = − 0.047, *p* = 0.689). Abbreviations: CSF, cerebrospinal fluid

In the total iNPH group, correlations among clinical scores and biomarkers were assessed using Spearman’s rank correlation. CSF and plasma p-Tau217 showed a strong positive correlation (ρ = 0.736, ***p < 0.001); however, the data distribution was highly skewed with dense clustering at lower values. By contrast, CSF and plasma AβO showed no significant correlation (ρ = − 0.047, *p* = 0.689) (Fig. 2). Plasma p-Tau217, in addition to its strong correlation with CSF p-Tau217, was inversely correlated with CSF Aβ42 (ρ = − 0.487, ***p < 0.001) and CSF Aβ42/40 ratio (ρ = − 0.337, **p = 0.003). Among clinical scores, plasma p-Tau217 correlated inversely with FAB (ρ = − 0.305, **p = 0.007) and positively with iNPH GS-D (ρ = 0.295, *p = 0.010), although effect sizes were weak. In contrast, plasma AβO showed generally low correlation coefficients with clinical scores and biomarkers, and even statistically significant correlations were of limited clinical relevance (Supplementary Table 1).

**Fig. 3:**
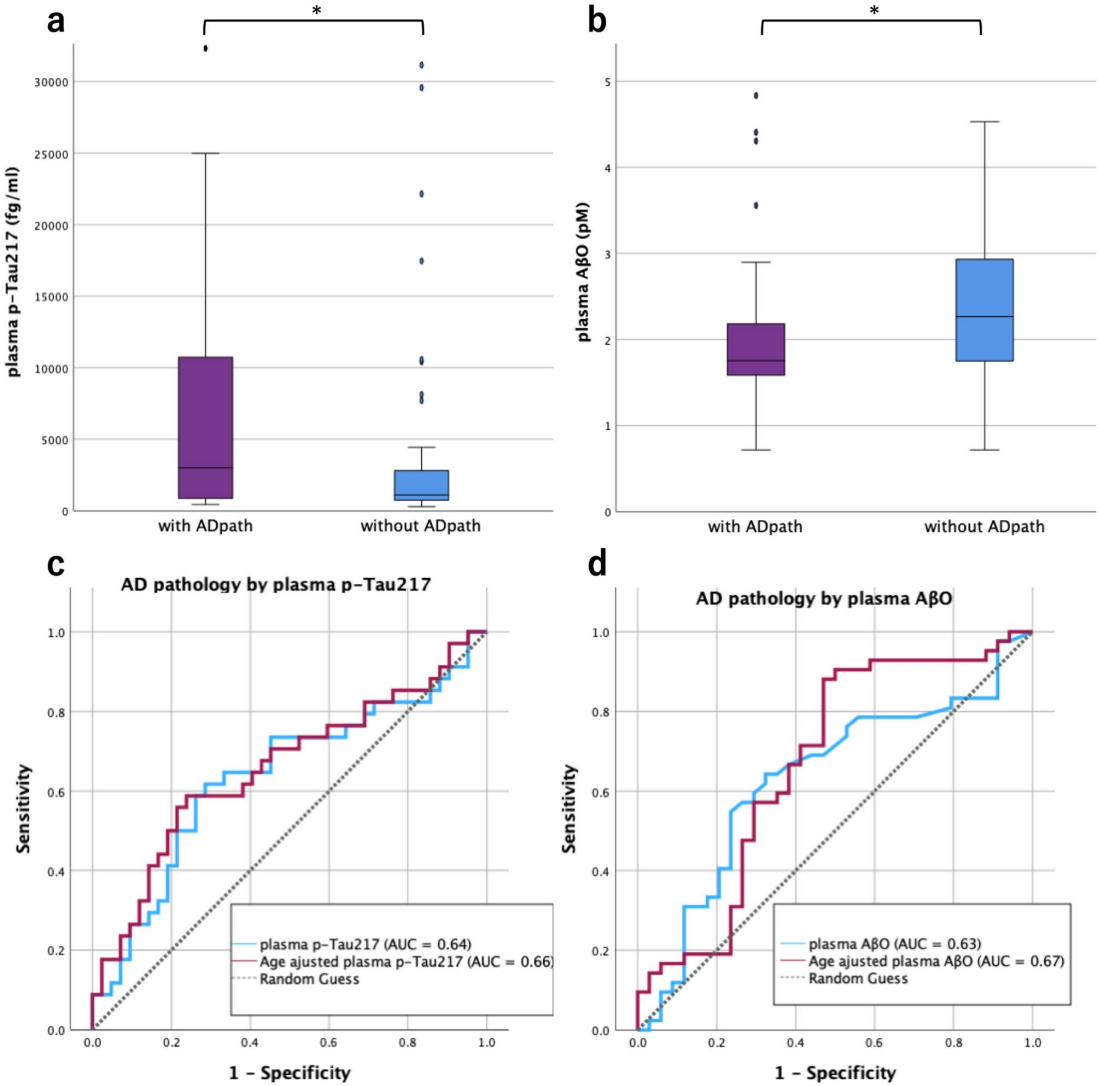
Box plots between the iNPH with and without AD pathology groups and ROC analyses for discrimination in the two groups by plasma biomarkers. Box-and-whisker plots show plasma p-Tau217 (**a**) and AβO (**b**) concentrations in the preoperative iNPH subgroups defined by CSF p‑Tau181 > 30 pg/mL: with AD pathology group (purple) and without AD pathology group (blue). Plasma p‑Tau217 levels were higher in the iNPH with AD pathology group (*p = 0.040), whereas plasma AβO levels were higher in the iNPH without AD pathology group (*p = 0.049). ROC curves show the discrimination to identify AD pathology in the preoperative iNPH group using plasma p‑Tau217 (**c**) and AβO (**d**). Blue lines indicate measured values and purple lines indicate age‑adjusted logistic models. The AUC values are shown in the in‑panel keys: p‑Tau217 0.64 (age‑adjusted 0.66); AβO 0.63 (age‑adjusted 0.67)

The iNPH group was stratified by CSF p-Tau181 using a cutoff of 30 pg/mL into iNPH with AD pathology (*n* = 34) and iNPH without AD pathology (*n* = 42). Age was significantly higher in the iNPH with AD pathology group (79.7 ± 6.2 vs. 76.7 ± 6.3 years, *p = 0.046). FAB was higher in the iNPH without AD pathology group (14 [13–16] vs. 12.5 [11–15], *p = 0.042), whereas MMSE showed no group difference. iNPH GS-D tended to be higher (worse) in the iNPH with AD pathology group, but the difference did not reach statistical significance (2 [1–3] vs. 1 [0–2], *p* = 0.056). Regarding biomarkers, plasma p-Tau217 (*p = 0.040), CSF Aβ40 (**p = 0.002), and CSF p-Tau217 (***p < 0.001) were significantly higher in the iNPH with AD pathology group, whereas plasma AβO was higher in the iNPH without AD pathology group (*p = 0.049) (Table 3; Fig. 3). In the additional comparison between the iNPH with AD pathology group and the AD group (Supplementary Table 2), demographic variables did not differ significantly between the iNPH with AD pathology group and the AD group, including age (*p* = 0.654) and sex distribution (*p* = 0.550). MMSE was significantly higher in the iNPH with AD pathology group than in the AD group (median [IQR]: 24 [21–28] vs. 16 [12–23.5], ***p < 0.001). Regarding plasma biomarker, plasma p-Tau217 was significantly elevated in the AD group compared with the iNPH with AD pathology group (15,963 ± 15,694 vs. 8,557 ± 12,077 fg/mL, **p = 0.005). Similarly, plasma AβO was also higher in the AD group (4.42 ± 3.38 vs. 2.07 ± 0.98 pM, **p = 0.006).

**Table 3:** Comparisons between the iNPH with and without AD pathology groups

	total iNPH (*n* = 76)	iNPH with AD pathology (*n* = 34)	iNPH without AD pathology (*n* = 42)	*p*-value
Age (mean ± SD)	78.0 ± 6.4	79.7 ± 6.2	76.7 ± 6.3	0.046*
Male (n, %)	42 (55.3%)	19 (55.9%)	23 (54.8%)	0.922
Evans index (mean ± SD)	0.345 ± 0.032	0.347 ± 0.032	0.343 ± 0.032	0.790
Clinical score (median [IQR])				
iNPH GS-G	2 [1–2]	2 [1–3]	2 [1–2]	0.198
iNPH GS-D	2 [1–3]	2 [1–3]	1 [0–2]	0.056
iNPH GS-U	1 [1–2]	1 [1–2]	1 [1–2]	0.969
total iNPH GS	4 [3–6]	5 [3–7]	4 [3–6]	0.133
MMSE	25 [22–28]	24 [21–28]	26 [22–29]	0.222
FAB	13 [11.25–16]	12.5 [11 − 15]	14 [13–16]	0.042*
CSF biomarker (mean ± SD)				
pTau181 (pg/mL)	35.9 ± 23.0	51.2 ± 27.1	23.5 ± 5.2	
Aβ42 (pg/mL)	627 ± 206	644 ± 208	612 ± 206	0.430
Aβ40 (pg/mL)	7722 ± 2344	8469 ± 1743	7117 ± 2601	0.002**
Aβ42/40	0.088 ± 0.036	0.078 ± 0.027	0.095 ± 0.041	0.077
p-Tau217 (fg/mL)	99,442 ± 136,582	165,479 ± 180,132	45,983 ± 38,263	< 0.001***
AβO (pM)	4.49 ± 3.16	4.87 ± 3.15	4.18 ± 3.16	0.284
Plasma biomarker (mean ± SD)				
p-Tau217 (fg/mL)	6188 ± 9970	8557 ± 12,077	4270 ± 7483	0.040*
AβO (pM)	2.27 ± 1.00	2.07 ± 0.98	2.43 ± 1.01	0.049*
p-Tau217/AβO ratio	3849 ± 8907	6396 ± 12,495	1787 ± 3120	0.019*

Baselines in the preoperative total iNPH group and pairwise comparison between the iNPH with and without AD pathology group are shown in table. *P*-values were calculated with Chi-square test (male) and Mann–Whitney U test (the others)

Because age differed significantly between the two groups, ROC analyses for plasma biomarkers were performed using both unadjusted values and logistic regression models adjusted for age (Table 4; Fig. 3). For plasma p-Tau217, the AUC was 0.64 (95% CI, 0.508–0.768), and the age-adjusted model yielded an AUC of 0.66 (95% CI, 0.531–0.785). The corresponding cutoff value of 1,963 fg/mL was associated with a sensitivity of 61.8% and a specificity of 71.4%. Plasma AβO showed an AUC of 0.63 (95% CI, 0.503–0.761), which increased to 0.67 (95% CI, 0.542–0.797) after age adjustment, with a cutoff of 1.94 pM (sensitivity 64.3%, specificity 67.6%). Similarly, the plasma p-Tau217/AβO ratio was significantly higher in the iNPH with AD pathology group (6,396 ± 12,495 vs. 1,787 ± 3,120, *p* = 0.019) (Table 3, Supplementary Fig. 2). The ROC analysis yielded an AUC of 0.66 (95% CI, 0.532–0.783), which increased to 0.68 (95% CI, 0.551–0.805) after age adjustment (Table 4, Supplementary Fig. 2).

**Table 4:** ROC analyses for AD pathology in iNPH by plasma biomarkers

	plasma *p*-Tau217 (fg/mL)	plasma AβO (pM)	plasma *p*-Tau217/AβO ratio
AUC			
measured value	0.64	0.63	0.66
age adjusted	0.66	0.67	0.68
95% CI			
measured value	0.508–0.768	0.503–0.761	0.532–0.783
age adjusted	0.531–0.785	0.542–0.797	0.551–0.805
Cutoff	1963	1.94	1141
Sensitivity	0.618	0.643	0.588
Specificity	0.714	0.676	0.714

ROC analyses for detecting the coexistence of AD pathology in the iNPH group are shown in table. Columns in the AUC and 95% CI summarize measured values (biomarker alone) and age‑adjusted logistic models (biomarker + age). Columns in the cutoff, sensitivity and specificity summarize measured values

Scatter plots of plasma p-Tau217 and AβO across the HCs, iNPH with and without AD pathology, and AD groups (Supplementary Fig. 3) demonstrated partial separation with substantial overlap: the AD cases tended to localize on the right with higher p‑Tau217, whereas the HCs and iNPH groups showed marked overlap; the iNPH with AD pathology group exhibited a modest rightward shift compared with the iNPH without AD pathology group.

### Study 3: Pre–post comparisons of clinical scores and biomarkers after shunt surgery

**Fig. 4:**
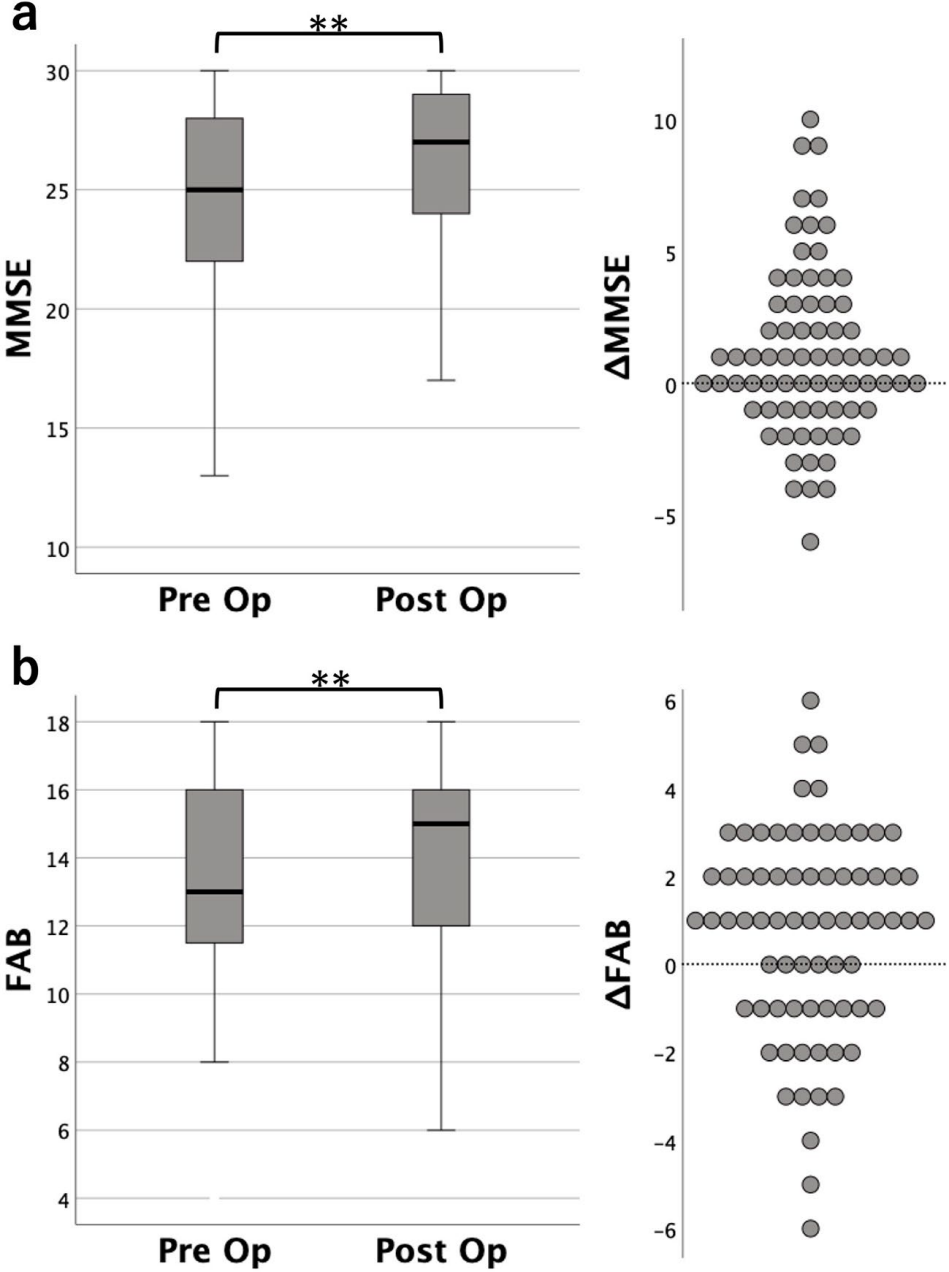
Pre–post changes in MMSE and FAB scores after shunt surgery in iNPH patients (a) MMSE and (b) FAB scores before and after shunt surgery. Boxplots represent median and interquartile range, with whiskers indicating the range of values. Preoperative and postoperative scores were compared using the Wilcoxon signed-rank test. Asterisks indicate statistical significance (**p < 0.01). The right panels show the distribution of individual change scores (Δ = postoperative – preoperative scores). Positive values indicate improvement for both MMSE and FAB

**Fig. 5:**
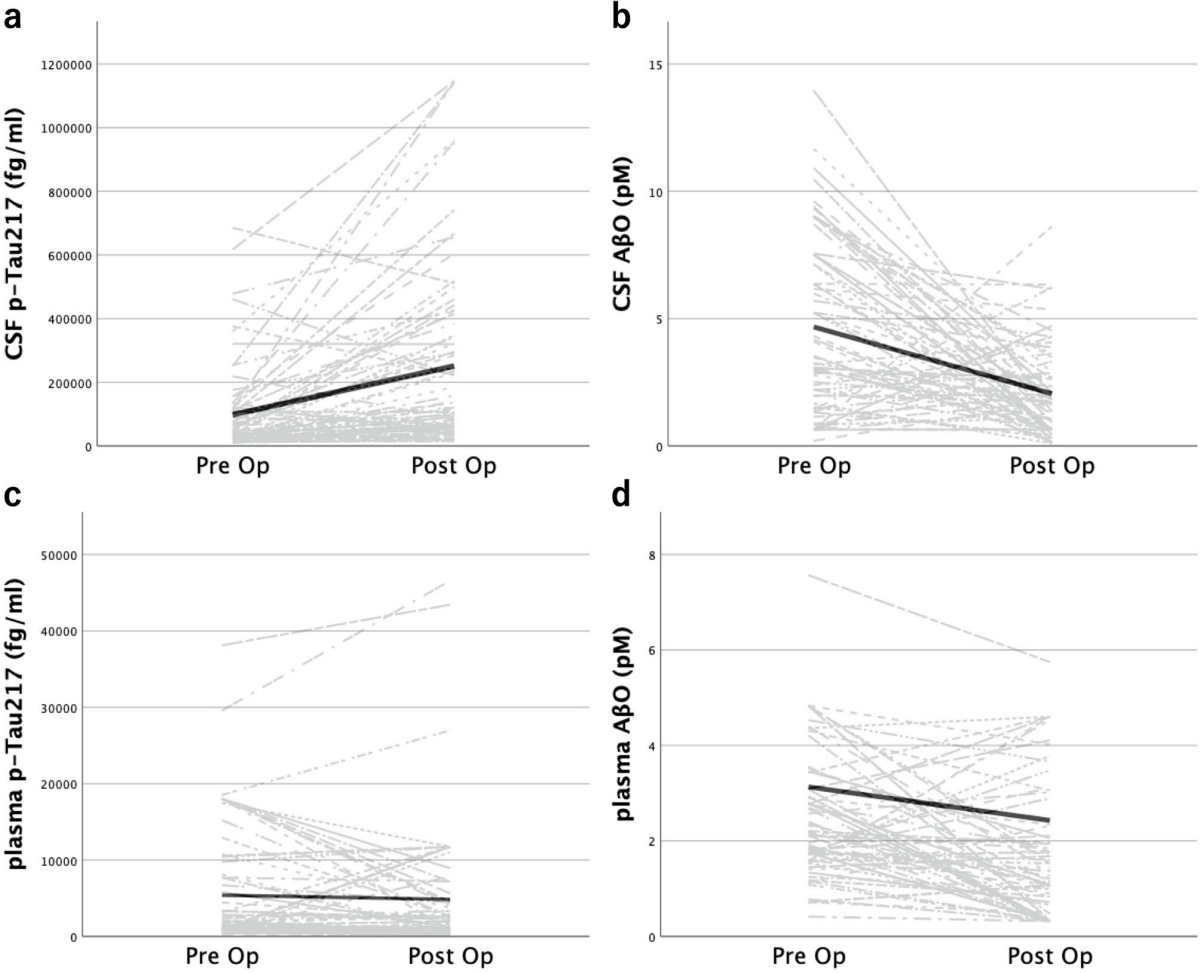
Individual pre–post changes in biomarkers after shunt surgery in iNPH patients. Panels show (**a**) CSF p-Tau217, (**b**) CSF AβO, (**c**) plasma p-Tau217, and (**d**) plasma AβO. Each gray line represents an individual patient, connecting preoperative and postoperative values, while black lines indicate the median change across patients. Preoperative and postoperative biomarker levels were compared using the Wilcoxon signed-rank test, as summarized in Table 5

In the total cohort of 75 cases, pre–post comparisons demonstrated significant improvements in iNPH GS, MMSE and FAB (Table 5; Fig. 4). Regarding biomarkers, CSF p-Tau217 significantly increased postoperatively (from 99,442 ± 136,582 fg/mL to 260,608 ± 315,152 fg/mL, ***p < 0.001), whereas CSF AβO significantly decreased (from 4.49 ± 3.16 pM to 2.10 ± 1.75 pM, ***p < 0.001). Plasma p-Tau217 showed no significant change, while plasma AβO significantly decreased after shunt surgery (from 2.27 ± 1.00 pM to 1.64 ± 1.31 pM, ***p < 0.001) (Table 5; Fig. 5). At the postoperative assessment, cognitive-related clinical scores (iNPH GS-D, MMSE, and FAB) were significantly better in the iNPH without AD pathology group than in the iNPH with AD pathology group (Table 6). Postoperative clinical assessments were performed at variable time points, ranging from 1 to 42 months after shunt surgery (mean ± SD, 10.0 ± 8.4 months). To evaluate whether postoperative changes differed between iNPH patients with and without concomitant AD pathology, we performed a two-way mixed repeated-measures ANCOVA with time (preoperative vs. postoperative) as the within-subject factor and AD pathology status (with vs. without) as the between-subject factor, including age as a covariate. The time × group interaction was not significant for any clinical outcome, including iNPH GS-G (*p* = 0.765, ηp² = 0.001), iNPH GS-D (*p* = 0.330, ηp² = 0.014), iNPH GS-U (*p* = 0.591, ηp² = 0.004), total iNPH GS (*p* = 0.968, ηp² < 0.001), MMSE (*p* = 0.159, ηp² = 0.029), and FAB (*p* = 0.665, ηp² = 0.003) (Supplementary Fig. 4). Although postoperative changes were observed in the expected direction in both groups, the magnitude of change did not differ significantly according to AD pathology status.

**Table 5:** Pre–post comparisons of clinical scores and biomarkers after shunt surgery

	Pre Op	Post Op	*p*-value
Clinical score (median [IQR])			
iNPH GS-G	2 [1–2]	1 [1–2]	< 0.001***
iNPH GS-D	2 [1–3]	1 [0–2]	< 0.001***
iNPH GS-U	1 [1–2]	1 [0–1]	0.002**
total iNPH GS	4 [3–6]	3 [2–5]	< 0.001***
MMSE	25 [22–28]	27 [24–29]	0.008**
FAB	13 [11.25–16]	15 [12–16]	0.006**
CSF biomarker (mean ± SD)			
p-Tau217 (fg/mL)	99,442 ± 136,582	260,608 ± 315,152	< 0.001***
AβO (pM)	4.49 ± 3.16	2.10 ± 1.75	< 0.001***
Plasma biomarker (mean ± SD)			
p-Tau217 (fg/mL)	6188 ± 9970	4178 ± 6840	0.062
AβO (pM)	2.27 ± 1.00	1.64 ± 1.31	< 0.001***

Pre–post comparisons of clinical scores and biomarkers before and after shunt surgery in the iNPH group are shown in table. Pre–post differences were calculated with Wilcoxon signed-rank test (two-sided). Significance in multiple comparison is shown as *p < 0.05; **p < 0.01; ***p < 0.001

**Table 6:** Comparisons of postoperative clinical scores between the iNPH with and without AD pathology groups

	iNPH with AD pathology (*n* = 34)	iNPH without AD pathology (*n* = 42)	*p*-value
Clinical score (median [IQR])			
post iNPH GS-G	2 [1–2]	1 [0–2]	0.153
post iNPH GS-D	2 [0–2]	1 [0–1]	0.019*
post iNPH GS-U	1 [0–1]	1 [0–2]	0.577
post total iNPH GS	4 [2–6]	3 [1.75–5]	0.102
post MMSE	25 [21–28]	27.5 [25.75–29]	0.020*
post FAB	14 [11–15]	15.5 [12.75–17]	0.032*

Postoperative clinical scores in the iNPH with vs. without AD pathology are shown in table. Between-group differences were calculated with Mann–Whitney U test (two-sided). Significance in multiple comparison is shown as *p < 0.05; **p < 0.01; ***p < 0.001

## Discussion

This study focused on plasma p-Tau217 and AβO, and examined three aspects: three-group comparisons among the HCs, iNPH, and AD groups (Study 1); correlation analyses between clinical scores and biomarkers of the iNPH group and stratification of iNPH by a cutoff of CSF p-Tau181 > 30 pg/mL with two-group comparisons (Study 2); longitudinal pre–post analyses after shunt surgery (Study 3). These results demonstrated that p-Tau217 sensitively reflects intracellular tau phosphorylation abnormalities and serves as a useful biomarker for distinguishing AD from non-AD. In contrast, AβO appears to capture amyloid pathology at relatively early stages and may function as a dynamic biomarker that varies with disease stage [9–13, 21].

### Complementary plasma biomarkers for iNPH

In study 1, plasma p-Tau217 clearly distinguished AD from iNPH and HCs, supporting its role as an AD-reflective biomarker. Notably, blood-based AD biomarker testing has rapidly expanded in clinical practice; for example, the U.S. Food and Drug Administration cleared a plasma p-Tau217/β‑amyloid 1‑42 ratio assay in May 2025 as an aid to identify amyloid pathology in symptomatic patients [22]. These developments underscore the potential role of plasma p‑Tau217–based testing as a practical screening/triage tool for evaluating concomitant AD pathology in iNPH, while emphasizing that it should be interpreted as an adjunct to clinical and imaging assessment rather than a stand‑alone diagnostic test. In contrast, plasma AβO increased stepwise from HCs to iNPH to AD, showed significant differences in all pairwise comparisons, and demonstrated moderate discriminative ability even between HCs and iNPH (AUC = 0.71). Considering the large variability observed within the AD group, and a previous report suggesting soluble AβO may decrease in advanced AD, AβO appears to be most sensitive at earlier stages [21]. The observed large variability in the plasma biomarker levels could potentially reflect differences in disease stage within the AD group. At the same time, the substantial overlap of individual values between HCs and iNPH indicates that its diagnostic utility at the individual level is limited. Importantly, AD-related pathology—particularly amyloid positivity—is common even among cognitively unimpaired older adults (e.g., > 40% amyloid positivity in those aged 80–89 years in a population-based cohort), and thus biomarker positivity should not be equated with a clinical diagnosis of AD without clinical correlation, including cognitive impairment [23]. Taken together, plasma p-Tau217 (for discrimination between AD and non-AD) and plasma AβO (for early-stage discrimination) may serve as complementary plasma biomarkers; however, further analyses with larger sample sizes are required to establish their clinical utility.

### Plasma biomarker correlations and iNPH stratification validity

In the preoperative iNPH group, plasma p-Tau217 showed a strong correlation with CSF p-Tau217 and also correlated with several clinical scores and other CSF biomarkers. However, this correlation should be interpreted with caution, as the data distribution was highly skewed, with a large proportion of samples clustered near the lower detection range, and the observed association may be partly influenced by a limited number of high-value observations. By contrast, plasma Aβ oligomer (AβO) levels showed little correlation with clinical scores or CSF biomarkers. This finding raises an important question regarding what plasma AβO concentrations actually represent. Unlike CSF biomarkers, which are generally considered to more directly reflect central nervous system pathology, plasma AβO likely represents a composite signal influenced by multiple sources and processes. These may include brain-derived Aβ species released into the circulation, peripheral production of amyloid peptides, and dynamic clearance mechanisms across the blood–brain barrier and peripheral organs such as the liver and kidney [9–11, 24–28]. Consequently, plasma AβO levels should not be interpreted as a simple surrogate of CSF AβO concentrations or cerebral amyloid burden. Furthermore, the weak correlation between plasma and CSF AβO may reflect the nonlinear and stage-dependent behavior of soluble Aβ oligomers [12, 13, 21]. Experimental and clinical studies suggest that AβO levels may increase during early disease stages and subsequently decline as oligomers aggregate into insoluble plaques or are sequestered within brain tissue. Such nonlinear kinetics, combined with impaired clearance pathways, could obscure linear associations between CSF and plasma compartments. Importantly, the associations observed in the present study do not imply a direct causal relationship between plasma AβO levels and clinical severity or central pathology. Rather, plasma AβO should be viewed as a downstream or integrative biomarker that may be modulated by systemic metabolism, blood–brain barrier transport, and cerebrospinal fluid dynamics, particularly in conditions such as iNPH where fluid circulation is altered.

Stratification using a cutoff of CSF p-Tau181 > 30 pg/mL was based on prior evidence that elevated preoperative p-Tau predicts less cognitive improvement after shunt surgery [17, 29–31]. Although this single threshold has limitations in iNPH due to the potential impact of CSF dynamics [5–7], the iNPH with AD pathology group showed consistent cross-sectional differences, including higher plasma p-Tau217, lower plasma AβO, higher CSF p-Tau217 and CSF Aβ40, and lower FAB, supporting the utility of CSF p-Tau181 as a provisional biomarker for the presence of AD pathology in iNPH.

Importantly, however, the additional comparison between the iNPH with AD pathology group and the AD group suggested that these two groups were not biologically equivalent. Despite the presence of AD pathology in both groups, plasma p-Tau217 and AβO levels were significantly lower in iNPH patients with AD pathology than in patients with AD alone, while cognitive impairment was less severe. These findings suggest that biomarker alterations observed in iNPH with AD pathology cannot be explained solely by the presence of AD-related pathology. Instead, they likely reflect disease-specific pathophysiological mechanisms unique to iNPH, such as impaired cerebrospinal fluid dynamics and altered clearance of AD-related proteins from the central nervous system to the peripheral circulation.

Regarding the discriminative ability of plasma biomarkers for the coexistence of AD pathology in the iNPH group, plasma p-Tau217 showed modest discrimination with an AUC of 0.64 and the effect of age adjustment was limited (AUC = 0.66). Plasma AβO was higher in the iNPH without AD pathology group; therefore, by applying a reverse evaluation designating “absence of AD pathology” as positive, the AUC was 0.63 (age-adjusted AUC = 0.67). The inverse pattern of higher p-Tau217 and lower AβO observed in the iNPH with AD pathology group may be explained by the decline in soluble AβO with disease progression and by alterations in BBB transport and sink/clearance mechanisms mediated by low-density lipoprotein receptor 1 (LRP1) efflux and receptor for advanced glycation end products (RAGE) influx [12, 13, 21, 24–27, 32]. Although the plasma p-Tau217/AβO ratio showed a slight improvement in discrimination (AUC = 0.66; age-adjusted AUC = 0.68), the diagnostic utility of these plasma biomarkers alone remained limited in the present study.

### Longitudinal biomarker changes and prediction

In this study, postoperative clinical scores showed significant improvement, and both CSF and plasma AβO decreased after shunt surgery. These findings were consistent with previous reports that AβO levels decline following shunting [13]. The association between postoperative clinical improvement and reduced AβO supports the hypothesis that enhanced CSF turnover promotes “sink/clearance” mechanisms [13, 18, 33]. Furthermore, the CSF-sink strategy proposes that reducing Aβ levels in enhances the gradient from the brain interstitial fluid to CSF, thereby promoting the clearance of brain amyloid. This mechanism is theoretically consistent with the effects of shunt [33]. The postoperative increase in CSF p-Tau217 can be interpreted as a phenomenon of increased detection due to enhanced efflux, dilution, or volume changes, rather than an immediate worsening of AD pathology. Plasma p-Tau217 remained largely stable, suggesting that peripheral dynamics of centrally derived p-Tau may be less influenced by short-term CSF changes [9–11, 24–26]. In this study, postoperative cognitive scores were better in the iNPH without AD pathology group, however an age-adjusted repeated-measures ANCOVA did not detect a significant time × AD pathology interaction across six clinical measures, and the corresponding interaction effect sizes were uniformly small (ηp² ≤ 0.029). In clinical practice, concomitant AD pathology is often assumed to attenuate the clinical responsiveness to shunt surgery in patients with iNPH [17, 29–31]. These findings suggest that, within the postoperative evaluation window of this study, the magnitude of short-term clinical change after shunt surgery did not differ detectably between iNPH patients with and without biomarker-defined AD pathology. Importantly, the absence of a significant interaction should not be interpreted as evidence of equivalence. The study may have been underpowered to detect small group differences, and some clinical scales may be subject to floor or ceiling effects. In addition, early postoperative improvement is likely to reflect reversible hydrocephalus-related components, whereas the impact of progressive neurodegenerative pathology may become more apparent over longer follow-up periods, particularly for cognitive outcomes. Therefore, longer-term longitudinal studies with larger sample sizes are warranted to clarify whether concomitant AD pathology influences the durability of symptomatic improvement and long-term cognitive trajectories after shunt surgery.

### Limitations and future directions

The limitations of this study include the sample size, variability in postoperative follow-up time points, the retrospective single-center design, and the lack of detailed and uniform AD staging information (e.g., A/T/N or positron emission tomography (PET)) within the AD group. In addition, the clinical scales used in this retrospective cohort have inherent limitations: MMSE and the iNPH grading scale are relatively coarse screening tools, and FAB primarily reflects frontal executive dysfunction and is not disease-specific for iNPH or AD. Regarding longitudinal CSF analyses, potential confounding by differences between lumbar and ventricular sampling sites and by rostro-caudal concentration gradients has been raised; however, all patients underwent lumbo‑peritoneal shunt surgery in our institution and CSF samples were obtained from the lumbar subarachnoid space both preoperatively and postoperatively, minimizing this concern. Finally, stratification by CSF p‑Tau181 > 30 pg/mL should be interpreted as a pragmatic [17, 29–31], outcome-oriented marker of concomitant AD pathology rather than as a strict A/T/N-based diagnostic classification. Future studies should accumulate larger case numbers, conduct analyses in multicenter, large-scale cohorts, and implement standardized longitudinal assessments (including disease staging and fixed postoperative time points) to further validate the utility of these biomarkers.

## Conclusions

This study is the first investigation of plasma p-Tau217 and AβO in iNPH. Taken together, these biomarkers, when applied in a complementary manner, may aid in distinguishing AD from iNPH and support the identification of concomitant AD pathology in iNPH, whereas discrimination between healthy controls and iNPH based on plasma biomarkers alone appears limited. Although plasma biomarkers showed potential to provide additional information on postoperative changes, their role in diagnostic screening and longitudinal monitoring should be interpreted cautiously and requires further validation in larger cohorts with longer follow-up periods.

## Electronic supplementary material

Below is the link to the electronic supplementary material.

Supplementary Material 1

Supplementary Material 2: Fig. 1. ROC analysis for discrimination in AD vs. HCs by plasma biomarkers. ROC curves show discrimination in AD vs. HCs using p‑Tau217 (blue) and AβO (purple). Curves are plotted as sensitivity versus 1–specificity; the diagonal grey dashed line indicates random guess. The AUC values are shown in the in‑panel keys: p‑Tau217 (0.88), AβO (0.82).

Supplementary Material 3: Fig. 2. Box plots between the iNPH with and without AD pathology groups and ROC analyses for discrimination in the two groups by plasma p-Tau217/AβO ratio. Box-and-whisker plots (a) show plasma p-Tau217/AβO ratio in the preoperative iNPH subgroups defined by CSF p‑Tau181 > 30 pg/mL: with AD pathology group (purple) and without AD pathology group (blue). The levels were higher in the iNPH with AD pathology group (*p = 0.019). ROC curves (b) show the discrimination to identify AD pathology in the preoperative iNPH group using plasma p-Tau217/AβO ratio. Blue lines indicate measured values and purple lines indicate age‑adjusted logistic models. The AUC values are shown in the in‑panel keys: measured values 0.66 (age‑adjusted 0.68).

Supplementary Material 4: Fig. 3. Scatter plots by plasma biomarkers. Scatter plots with log10-transformed scales for both axes illustrate the distribution of individual cases across diagnostic groups: healthy controls (HCs; green circles), iNPH without AD pathology (blue triangles), iNPH with AD pathology (red triangles), and Alzheimer’s disease (AD; red circles). Grey vertical and horizontal lines indicate reference cut-off values derived from ROC analyses.

Supplementary Material 5: Fig. 4. Age-adjusted pre- and postoperative clinical scores in the iNPH with and without AD pathology groups. Age-adjusted estimated marginal means of clinical scores before and after shunt surgery in the iNPH with and without AD pathology groups. (a) iNPH GS-G, (b) iNPH GS-D, (c) iNPH GS-U, (d) total iNPH GS, (e) MMSE, and (f) FAB. Points and lines represent estimated marginal means from a two-way mixed repeated-measures ANCOVA with age as a covariate; error bars indicate 95% confidence intervals. P values and partial eta-squared (ηp²) shown in each panel correspond to the time (preoperative vs. postoperative) × group (with vs. without AD pathology) interaction. Lower iNPH grading scale scores indicate better symptom severity, whereas higher MMSE and FAB scores indicate better cognitive performance. Abbreviations: iNPH GS, iNPH grading scale gait; MMSE, Mini Mental State Examination; FAB, Frontal Assessment Battery; ANCOVA, analysis of covariance.

## Data Availability

The datasets generated and/or analyzed during the current study are available from the corresponding author on reasonable request.

## Abbreviations

iNPH: Idiopathic normal pressure hydrocephalus
CSF: Cerebrospinal fluid
AD: Alzheimer’s disease
p-Tau217: Phosphorylated tau 217
AβO: Amyloid-β oligomers
HCs: Healthy controls
iNPH GS: iNPH grading scale
NIA-AA: National Institute on Aging and Alzheimer’s Association
MMSE: Mini Mental State Examination
ROC: Receiver operating characteristic
FAB: Frontal Assessment Battery
LPS: Lumbo‑peritoneal shunt
AUC: Area under the curve
LRP1: Low-density lipoprotein receptor 1
RAGE: Receptor for advanced glycation end products
PET: Positron emission tomography
