# Supplementary figures and images for: Plasma p-Tau217 and amyloid-β oligomers as complementary biomarkers for differential diagnosis, comorbidity detection and disease monitoring in idiopathic normal pressure hydrocephalus
Source: Fluids Barriers CNS. 2026 Mar 5;23:59. doi: 10.1186/s12987-026-00784-8 (PMC13077803; doi:10.1186/s12987-026-00784-8)

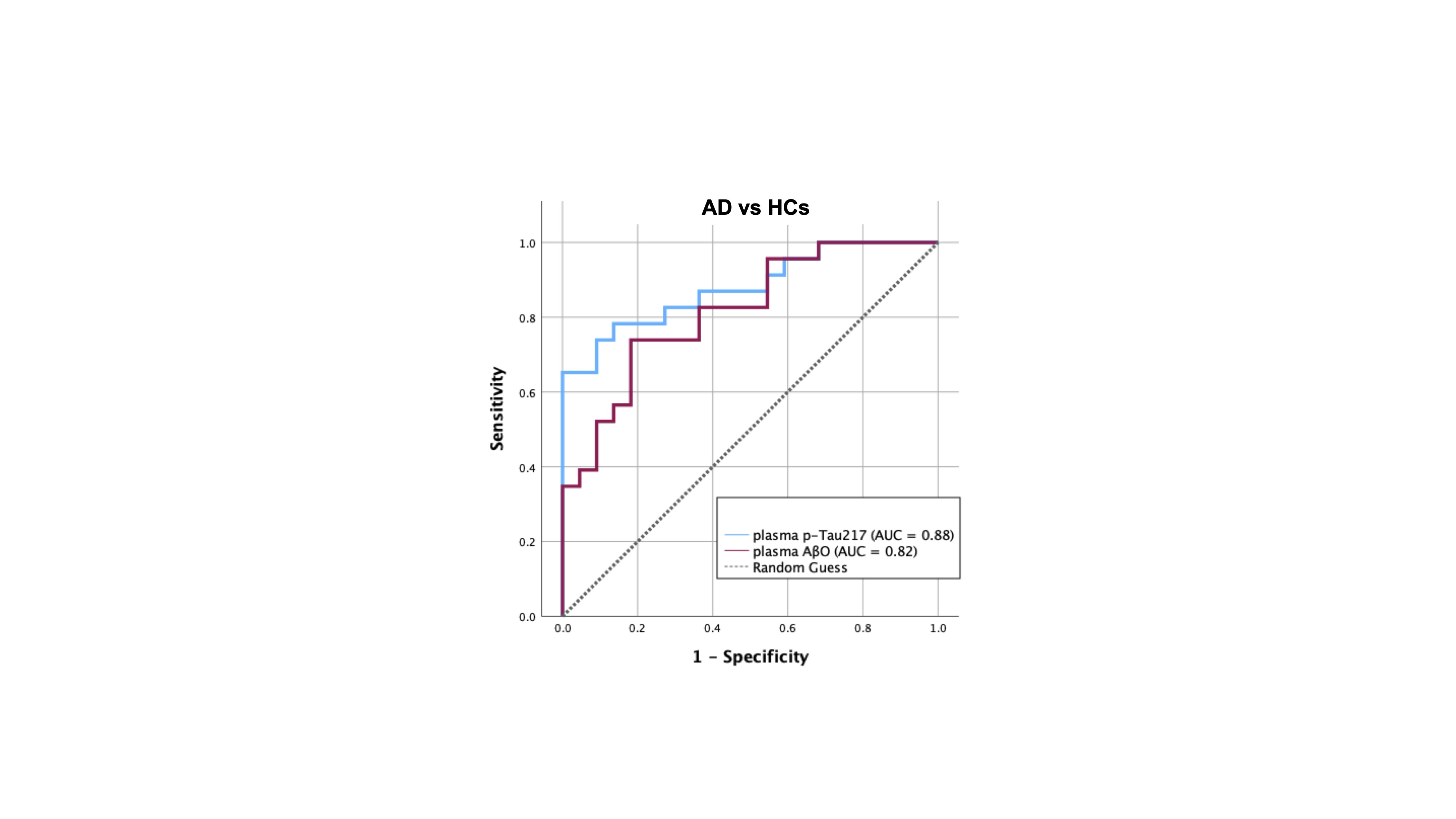

Supplement: Supplementary file 2 — Supplementary Material 2: Fig. 1. ROC analysis for discrimination in AD vs. HCs by plasma biomarkers. ROC curves show discrimination in AD vs. HCs using p‑Tau217 (blue) and AβO (purple). Curves are plotted as sensitivity versus 1–specificity; the diagonal grey dashed line indicates random guess. The AUC values are shown in the in‑panel keys: p‑Tau217 (0.88), AβO (0.82). [file 12987_2026_784_MOESM2_ESM.jpg]

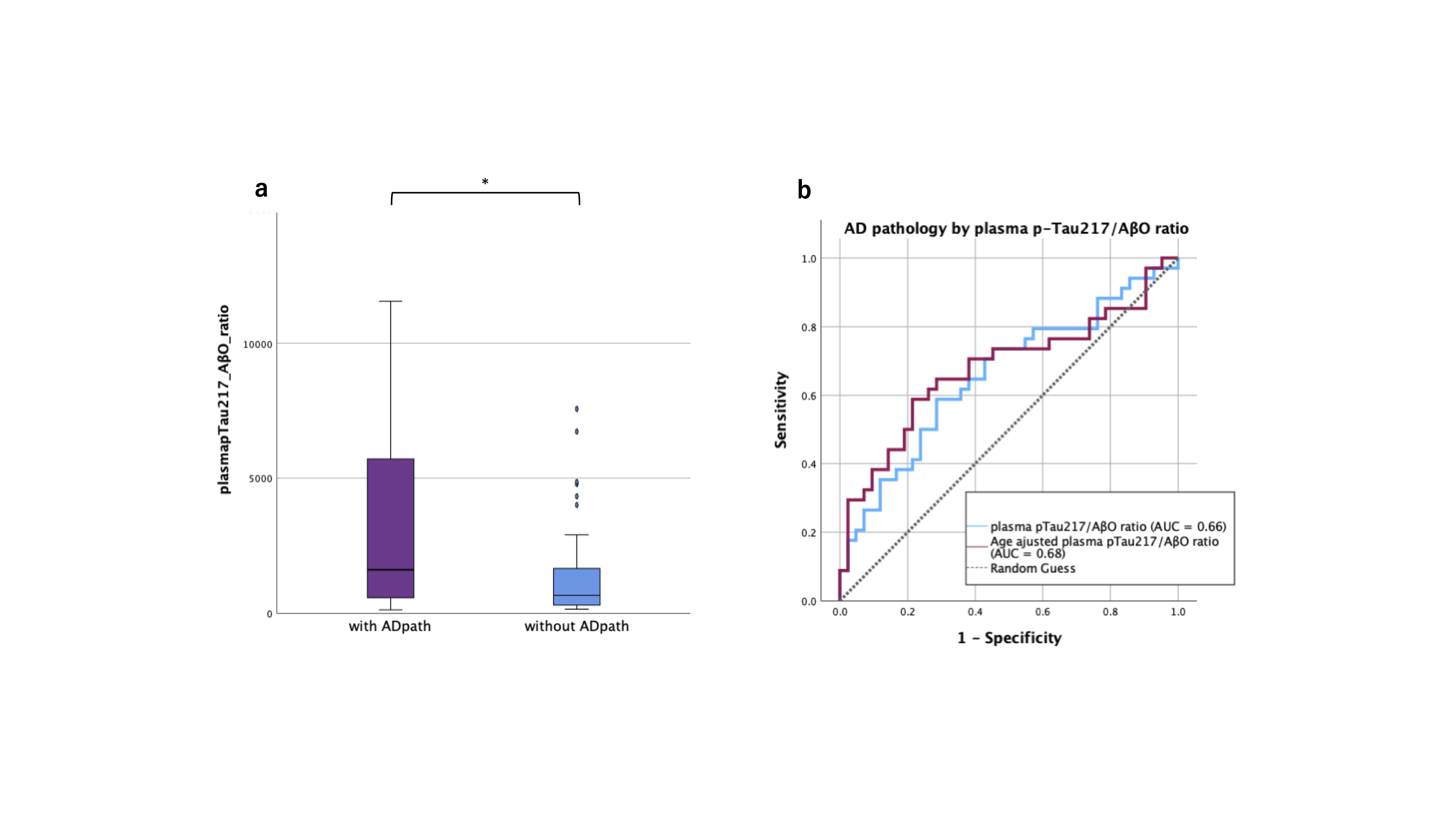

Supplement: Supplementary file 3 — Supplementary Material 3: Fig. 2. Box plots between the iNPH with and without AD pathology groups and ROC analyses for discrimination in the two groups by plasma p-Tau217/AβO ratio. Box-and-whisker plots (a) show plasma p-Tau217/AβO ratio in the preoperative iNPH subgroups defined by CSF p‑Tau181 > 30 pg/mL: with AD pathology group (purple) and without AD pathology group (blue). The levels were higher in the iNPH with AD pathology group (*p = 0.019). ROC curves (b) show the discrimination to identify AD pathology in the preoperative iNPH group using plasma p-Tau217/AβO ratio. Blue lines indicate measured values and purple lines indicate age‑adjusted logistic models. The AUC values are shown in the in‑panel keys: measured values 0.66 (age‑adjusted 0.68). [file 12987_2026_784_MOESM3_ESM.jpg]

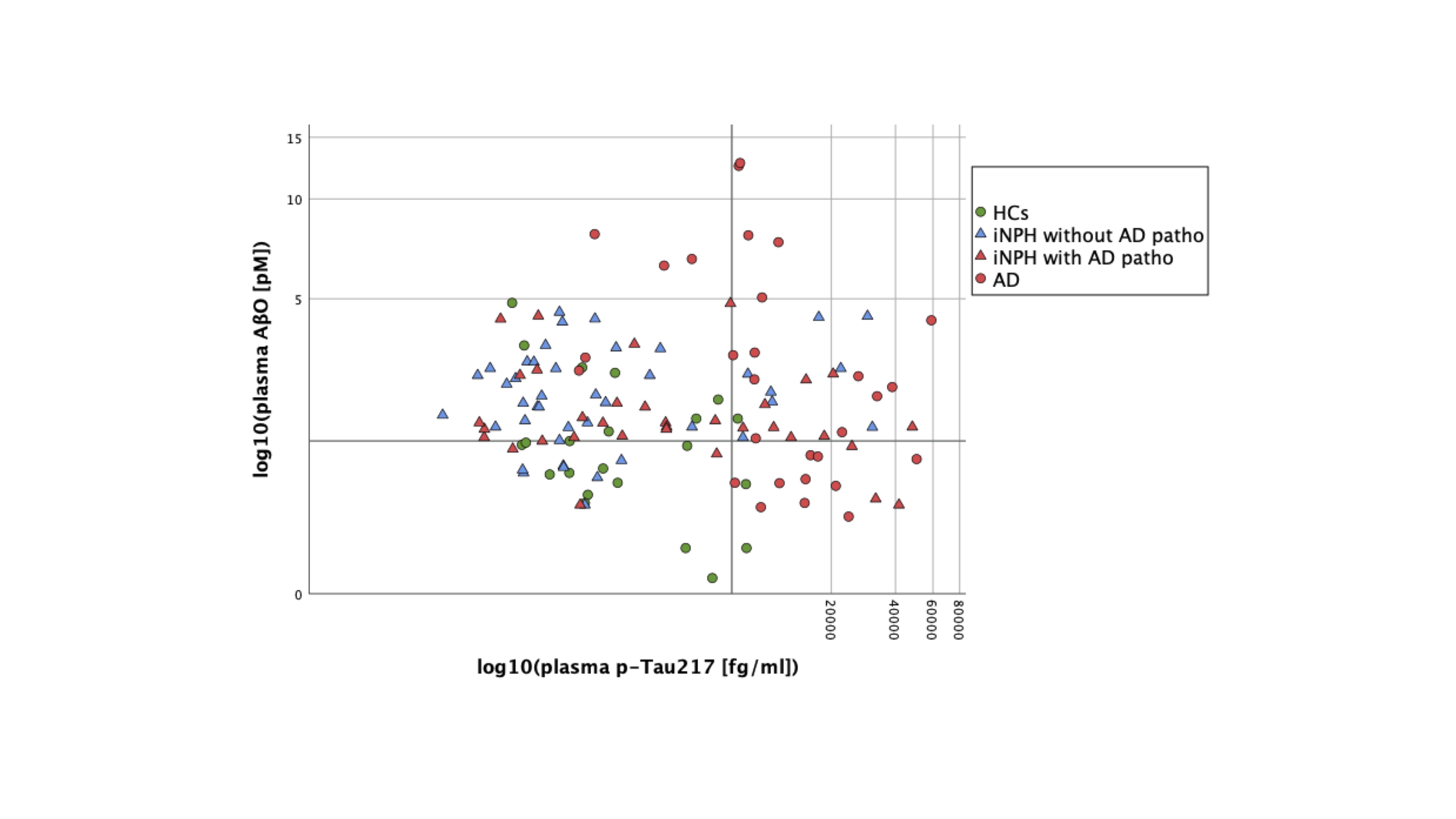

Supplement: Supplementary file 4 — Supplementary Material 4: Fig. 3. Scatter plots by plasma biomarkers. Scatter plots with log10-transformed scales for both axes illustrate the distribution of individual cases across diagnostic groups: healthy controls (HCs; green circles), iNPH without AD pathology (blue triangles), iNPH with AD pathology (red triangles), and Alzheimer’s disease (AD; red circles). Grey vertical and horizontal lines indicate reference cut-off values derived from ROC analyses. [file 12987_2026_784_MOESM4_ESM.jpg]

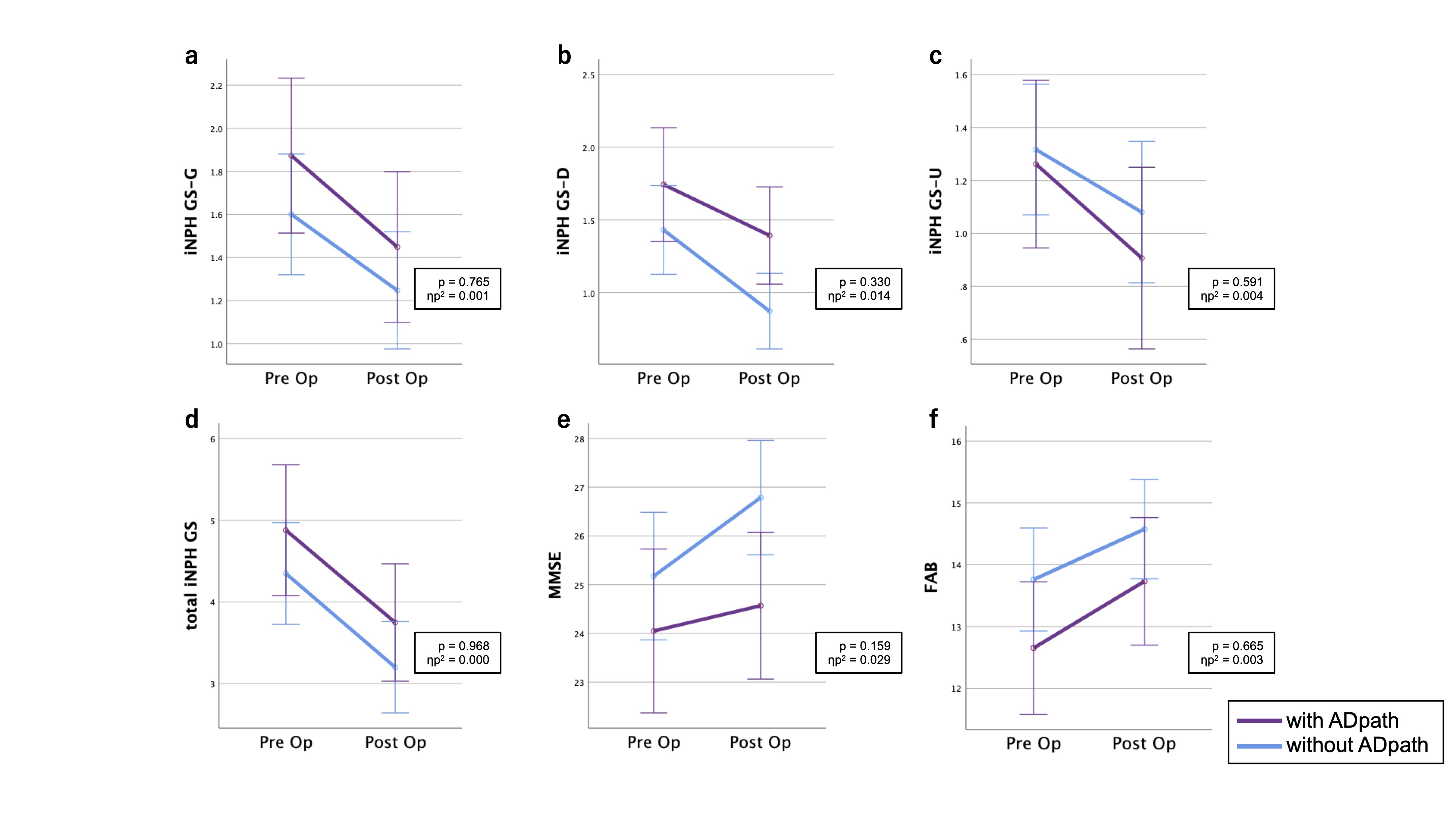

Supplement: Supplementary file 5 — Supplementary Material 5: Fig. 4. Age-adjusted pre- and postoperative clinical scores in the iNPH with and without AD pathology groups. Age-adjusted estimated marginal means of clinical scores before and after shunt surgery in the iNPH with and without AD pathology groups. (a) iNPH GS-G, (b) iNPH GS-D, (c) iNPH GS-U, (d) total iNPH GS, (e) MMSE, and (f) FAB. Points and lines represent estimated marginal means from a two-way mixed repeated-measures ANCOVA with age as a covariate; error bars indicate 95% confidence intervals. P values and partial eta-squared (ηp²) shown in each panel correspond to the time (preoperative vs. postoperative) × group (with vs. without AD pathology) interaction. Lower iNPH grading scale scores indicate better symptom severity, whereas higher MMSE and FAB scores indicate better cognitive performance. Abbreviations: iNPH GS, iNPH grading scale gait; MMSE, Mini Mental State Examination; FAB, Frontal Assessment Battery; ANCOVA, analysis of covariance. [file 12987_2026_784_MOESM5_ESM.jpg]
